# Multispectral Bioluminescence Tomography: Methodology and Simulation

**DOI:** 10.1155/IJBI/2006/57614

**Published:** 2006-02-05

**Authors:** Alexander X. Cong, Ge Wang

**Affiliations:** Bioluminescence Tomography Laboratory, Department of Radiology, University of Iowa, Iowa City, IA 52242, USA

## Abstract

Bioluminescent imaging has proven to be a valuable tool for monitoring physiological and pathological activities at cellular and molecular levels in living small animals. Using biological techniques, target cells can be tagged with reporters encoding several kinds of luciferase enzymes, which generate characteristic photons in a wide spectrum covering the infrared range. Part of the diffused light can reach the body surface of the small animal, be separated into several spectral bands using appropriate filters, and collected by a sensitive CCD camera. Here we present a bioluminescence tomography (BLT) method for a bioluminescent source reconstruction from multispectral data measured on the external surface, and demonstrate the advantages of multispectral BLT in a numerical study using a heterogeneous mouse chest phantom. The results show that the multispectral approach significantly improves the accuracy and stability of the BLT reconstruction even if the data are highly noisy.


Hindawi Publishing Corporation
International Journal of Biomedical Imaging
Volume 2006, Article ID 57614, Pages 1–7
DOI 10.1155/IJBI/2006/57614

### Multispectral Bioluminescence Tomography: Methodology and Simulation

**Alexander X. Cong and Ge Wang**

*Bioluminescence Tomography Laboratory, Department of Radiology, University of Iowa, Iowa City, IA 52242, USA*

Received 18 September 2005; Accepted 11 November 2005

Recommended for Publication by Jie Tian

Bioluminescent imaging has proven to be a valuable tool for monitoring physiological and pathological activities at cellular and molecular levels in living small animals. Using biological techniques, target cells can be tagged with reporters encoding several kinds of luciferase enzymes, which generate characteristic photons in a wide spectrum covering the infrared range. Part of the diffused light can reach the body surface of the small animal, be separated into several spectral bands using appropriate filters, and collected by a sensitive CCD camera. Here we present a bioluminescence tomography (BLT) method for a bioluminescent source reconstruction from multispectral data measured on the external surface, and demonstrate the advantages of multispectral BLT in a numerical study using a heterogeneous mouse chest phantom. The results show that the multispectral approach significantly improves the accuracy and stability of the BLT reconstruction even if the data are highly noisy.

Copyright © 2006 A. X. Cong and G. Wang. This is an open access article distributed under the Creative Commons Attribution License, which permits unrestricted use, distribution, and reproduction in any medium, provided the original work is properly cited.

### 1. INTRODUCTION

Bioluminescent imaging (BLI) is an emerging technology to monitor molecular and cellular activities in vivo using various small animal models. This new modality is extremely sensitive, cost-effective, and nontoxic for investigating a wide variety of diseases such as cancers and facilitating drug development [1–3]. Bioluminescence tomography (BLT) is a major frontier of BLI.

In bioluminescent imaging, target cells are labeled with reporter genes encoding luciferase enzymes in a living small animal. Upon a chemical reaction with a substrate luciferin in the presence of ATP and oxygen, the luciferase releases photons to allow observation of molecular and cellular activities [2]. Luciferase enzymes from firefly (Fluc), click beetle (CBGr68, CBRed), and *Renilla reniformis* (hRluc) are often utilized as reporter genes. It was shown that these four kinds of luciferase enzymes have different emission spectra: at temperature 37°C, Fluc, CBGr68, CBRed, and hRLuc exhibit their spectral peaks of 612 nm, 543 nm, 615 nm, and 480 nm, respectively [4]. Recently, a tricolor reporter was also developed, which emits green to red light [5]. These results enable multispectral BLT and its biomedical applications.

The bioluminescent photon propagation in the biological tissue is subject to both scattering and absorption. A significant number of bioluminescent photons escape from the body surface of the animal [6]. Using optical filters of different spectral bands, the photons in each spectral band can be captured by a highly sensitive CCD camera. In any band, the photon propagation in the tissue is typically described by the radiative transport equation [7]. However, a direct solution to the transport equation is not practically affordable due to the computational complexity. Since the scattering predominates over the absorption in this context, the diffusion model can be used as a good approximation to the physical process [8]. With filter techniques, the optical properties of the tissue can be determined for every spectral band using diffuse reflectance measurements [9, 10]. Diffuse optical tomography (DOT) can also be applied to reconstruct the band-specific spatially variable optical parameters [11]. Then, based on the diffusion approximation, the BLT can be formulated as an inverse source problem. This problem is severely underdetermined and ill-posed. Recently, Wang et al. described the BLT principles [12, 13] and reported the uniqueness of the solution dependent on a priori knowledge [14]. Several reconstruction methods for BLT were reported with numerical and phantom data [15–20]. The methods were found to perform well when a priori knowledge such as permissible source region was specified to overcome the ill-posedness of the problem.

In this paper, we present a multispectral approach to enhance the accuracy and stability of the BLT reconstruction. To avoid any confusion, we would underline that there can be two meanings attached to the concept of multispectral BLT. The first interpretation is that if we have only a single bioluminescent source that is spatially and spectrally distributed we can decompose its spectrum into a number of bands or channels for bandwise measurement, and then perform the source reconstruction. The second interpretation is that when we have multiple bioluminescent sources that are spatially and spectrally distributed independently, we can decompose their spectra respectively for according measurement and reconstruct all these generally different source distributions in an integrated fashion. As an initial investigation to investigate multispectral BLT, we assume the first interpretation of the multispectral BLT approach. Alternatively, we may also refer to our method as a multiband BLT algorithm in this context.

In this project, multiple sets of surface data corresponding to wavelength bands are measured to help improve the BLT reconstruction significantly. A finite-element discretization of the diffusion approximation model is utilized to establish a linear relationship between the source photon density and the measured surface photon density in each band. A linear least square optimization is performed for multispectral BLT reconstruction. In the next section, we will present the multispectral BLT methodology. Then, we will demonstrate the feasibility of our method in numerical simulation. In the last section, we will discuss the relevant issues and draw a conclusion.

### 2. METHODOLOGY

### 2.1. Diffusion approximation

It was reported that the range of light emission peaks for characterized luciferase enzymes is 460–630 nm [4]. In this spectral range, the photons are heavily scattered in the tissue, and the diffusion approximation is quite appropriate to describe the photon propagation [8]. The spectrum can be divided into a number of bands $[w_\nu, w_{\nu+1}]$, $\nu = 1, 2, \ldots, \tau$. In each spectral band $[w_\nu, w_{\nu+1}]$, the diffusion equation can be applied independently:

$$-\nabla \cdot \left(D_\nu(\mathbf{r})\nabla\Phi_\nu(\mathbf{r})\right) + \mu_{\nu a}(\mathbf{r})\Phi_\nu(\mathbf{r}) = S_\nu(\mathbf{r}), \quad \nu = 1, 2, \ldots, \tau \quad (\mathbf{r} \in \Omega), \tag{1}$$

where $D_\nu(\mathbf{r}) = (3(\mu_{\nu a}(\mathbf{r}) + \mu'_{\nu s}(\mathbf{r})))^{-1}$, $\Phi_\nu(\mathbf{r})$ is the photon density within $[w_\nu, w_{\nu+1}]$, $S_\nu(\mathbf{r})$ the photon density of a bioluminescent source within $[w_\nu, w_{\nu+1}]$, $\mu_{\nu a}(\mathbf{r})$ the absorption coefficient within $[w_\nu, w_{\nu+1}]$, and $\mu'_{\nu s}(\mathbf{x})$ the corresponding reduced scattering coefficient. Since our bioluminescent imaging experiments are performed in a dark environment, little external photons enter $\Omega$ through its boundary $\partial\Omega$. Taking into account the mismatch between the refractive indices $\gamma$ for $\Omega$ and $\gamma'$ for the surrounding medium, the boundary condition is expressed as [21, 22]

$$\Phi_\nu(\mathbf{r}) + 2A(\mathbf{r})D_\nu(\mathbf{r})\left(n \cdot \nabla\Phi_\nu(\mathbf{r})\right) = 0 \quad (\mathbf{r} \in \partial\Omega), \tag{2}$$

where $n$ is the unit outer normal to $\partial\Omega$, and $A(\mathbf{r}) = (1 + R(\mathbf{r}))/(1 - R(\mathbf{r}))$; $R(\mathbf{r})$ depends on the refractive index $\gamma$ of the medium, where $R(\mathbf{r}) \approx -1.4399\gamma^{-2} + 0.7099\gamma^{-1} + 0.6681 + 0.0636\gamma$. Finally, with the emission filter of bandpass $[w_\nu, w_{\nu+1}]$ the measured quantity is the outgoing flux density on $\partial\Omega$ [22]:

$$Q_\nu(\mathbf{r}) = -D_\nu(\mathbf{r})\left(n \cdot \nabla\Phi_\nu(\mathbf{r})\right) = \frac{1}{2A(\mathbf{r})}\Phi_\nu(\mathbf{r}), \quad \nu = 1, 2, \ldots, \tau \quad (\mathbf{r} \in \partial\Omega). \tag{3}$$

### 2.2. Image reconstruction

For each spectral band, the diffusion equation (1) and its boundary condition (2) can be formulated into a matrix equation using the finite-element method as follows [23]:

$$\left([\mathbf{K}_\nu] + [\mathbf{C}_\nu] + [\mathbf{B}_\nu]\right)\{\Phi_\nu\} = [\mathbf{F}_\nu]\{S_\nu\}, \quad \nu = 1, 2, \ldots, \tau, \tag{4}$$

where $\{\Phi_\nu\}$ and $\{S_\nu\}$ are the collection of all the nodal values of the photon density $\Phi_\nu(\mathbf{r})$ and source density $S_\nu(\mathbf{r})$, respectively. Let $[\mathbf{M}_\nu] \doteq ([\mathbf{K}_\nu] + [\mathbf{C}_\nu] + [\mathbf{B}_\nu])$, where $[\mathbf{M}_\nu]$ is a positive-definite matrix. Then, the photon density $\{\Phi_\nu\}$ can be obtained from (4):

$$\{\Phi_\nu\} = [\mathbf{M}_\nu]^{-1}[\mathbf{F}_\nu]\{S_\nu\}, \quad \nu = 1, 2, \ldots, \tau. \tag{5}$$

The reconstruction of the bioluminescent source is to identify the vector $\{S_\nu\}$ from the photon density $\{\Phi_\nu^{\text{meas}}\}$ measured on the side surface. Clearly, $\{\Phi_\nu\}$ can be partitioned into measurable boundary data $\{\Phi_\nu^{\text{meas}}\}$ and interior values $\{\Phi_\nu^{\text{intr}}\}$ that cannot be obtained from the measurement data. To regularize the BLT reconstruction, we should incorporate a priori knowledge obtained from bioluminescent measurement as well as biomedical, physiological, and anatomical information. As a result, the vector $\{S_\nu\}$ can be divided into two parts: $\{S_\nu^p\}$ and $\{S_\nu^0\}$. $\{S_\nu^p\}$ corresponds to the permissible region $\Omega_p$ where a bioluminescence source may reside, while $\{S_\nu^0\}$ corresponds to the forbidden region $\Omega_0$ without any bioluminescence source. $\{S_\nu^0\}$ vanishing is equivalent to removing those columns of $[\mathbf{B}_\nu] = [\mathbf{M}_\nu]^{-1}[\mathbf{F}_\nu]$, $\nu = 1, 2, \ldots, \tau$, that correspond to the vector $\{S_\nu^0\}$. Then, a linear relationship between the measurable photon density $\{\Phi_\nu^{\text{meas}}\}$ and the source density $\{S_\nu^p\}$ is established by deleting those rows of $[\mathbf{B}_\nu]$ that correspond to $\{\Phi_\nu^{\text{intr}}\}$ to obtain $[\overline{\mathbf{B}}_\nu]$:

$$\{\Phi_\nu^{\text{meas}}\} = [\overline{\mathbf{B}}_\nu]\{S_\nu^p\}, \quad \nu = 1, 2, \ldots, \tau. \tag{6}$$

By performing a spectral analysis, the energy contribution of a bioluminescent source can be determined over the entire spectral interval [4] as represented by $S_\nu^p = \omega_\nu S^t$, where $\omega_\nu \geq 0$ and $\sum_{\nu=1}^{\tau} \omega_\nu \approx 1$, $S^t$ denotes the total photon density, and $S_\nu^p$ the photon density within $[w_\nu, w_{\nu+1}]$, as shown in Figure 1. From the spectral distribution of a bioluminescence source and (6), the following linear system is obtained:

$$[\mathbf{B}]\{S^t\} = \{\Phi^{\text{meas}}\}, \tag{7}$$

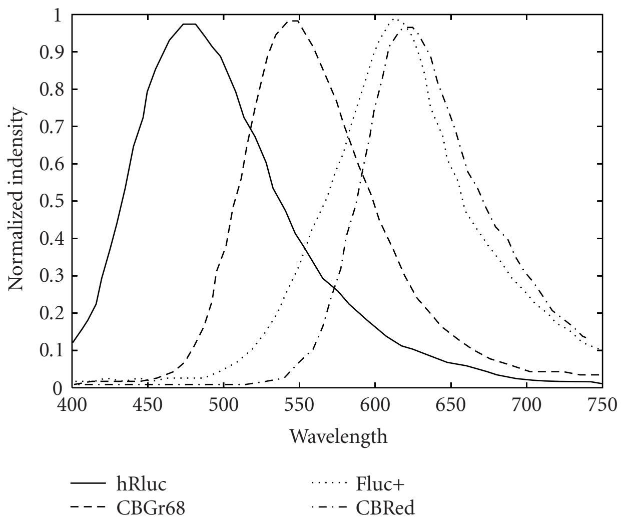

Figure 1: Spectral distributions of luciferase enzymes measured using spectrometry (modified from [4] with permission from the journal and the authors).

where

$$[\mathbf{B}] \doteq \begin{bmatrix} \omega_1\overline{\mathbf{B}}_1 \\ \omega_2\overline{\mathbf{B}}_2 \\ \vdots \\ \omega_\tau\overline{\mathbf{B}}_\tau \end{bmatrix}, \qquad \{\Phi^{\text{meas}}\} \doteq \begin{bmatrix} \Phi_1^{\text{meas}} \\ \Phi_2^{\text{meas}} \\ \vdots \\ \Phi_\tau^{\text{meas}} \end{bmatrix}. \tag{8}$$

Because the measured bioluminescent data are typically corrupted by noise, it is not optimal to solve for $\{S^t\}$ directly from (7). Hence, we propose to use the following optimization procedure to find a regularized solution [24]:

$$\min_{0\le S_i^t \le U_\nu} \{||[\mathbf{B}]\{S^t\} - \{\Phi^{\text{meas}}\}||_\Lambda + \alpha\eta(\{S^t\})\}, \tag{9}$$

where $U_\nu$ stands for an upper bound, $S_i^t$ the values in $\{S^t\}$, $\Lambda$ a weight matrix, $\|\mathbf{V}\|_\Lambda = \mathbf{V}^{\mathbf{T}}\mathbf{\Lambda}\mathbf{V}$, $\eta$ a stabilizing function, and $\alpha$ the regularization parameter. This is a standard linear least square problem with constrains. From (7), the source densities $S^t$ are constrained by the measured multispectral information, which helps improve the stability of the reconstruction.

### 3. NUMERICAL SIMULATION

### 3.1. Spectral distributions

Bioluminescent spectral analysis experiment [4] shows that the four kinds of luciferase enzymes hRLuc, CNGr68, Fluc+, and CBRed have different emission peaks at 480 nm, 543 nm, 612 nm, and 615 nm, respectively, as shown in Figure 1. Their fusion creates broad emission spectra. The spectral properties of the luciferase light source should have a significant impact on how much of the light is transmitted to the external surface. The detected polychromatic photons carry more information about the bioluminescence source than the corresponding integrated signals. We used the experimental data in Figure 1 to estimate the energy distribution for our numerical simulation. Let us assume that target cells be tagged with reporters encoded with the four kinds of luciferase enzymes and that the cells emit photons in the spectral range [400 nm, 750 nm]. Based on the emission spectral distribution, the spectrum may be divided into the following three regions: [400 nm, 530 nm], [530 nm, 630 nm], and [630 nm, 750 nm]. By integrating the intensity over each spectral region, we can quantify the energy distribution as $\omega_3 = 0.29$ for [400 nm, 530 nm], $\omega_2 = 0.48$ for [530 nm, 630 nm], and $\omega_3 = 0.23$ for [630 nm, 750 nm].

### 3.2. Single-band reconstruction

The numerical simulation was performed using a heterogeneous numerical phantom that contains regions resembling lungs, heart, muscle, and bone. This cylindrical phantom had a diameter of 30 mm and height of 26 mm, as shown in Figure 2. The phantom was discredited into 6576 vertex nodes and 11340 prism elements. The two bioluminescent sources were embedded in the left lung, as shown in Figure 3(a). The first source was located at (−8.66, 3.46, 13.1), while the second one at (−10.21, −3.17, 13.1). Both sources had photon density of 300 pW/mm$^3$. The permissible region was selected based on a priori knowledge, as shown in Figure 3(b). This region contained 308 elements. The optical parameters averaged over the spectral range [400 nm, 750 nm] for each type of structure in the heterogeneous phantom are listed in Table 1 [16, 18].

The simulated measurement data on the 1024 detector points on the phantom side surface were generated according to the finite-element forward model. Then, the measurement data were corrupted with 20% Gaussian noise to simulate measurement errors. We set the stabilizing function $\eta(X) = X^T X$ and the regularization parameter $\alpha = 3.0 \times 10^{-8}$, and then performed the source reconstruction using our published single-band BLT algorithm [18]. The reconstructed locations of the sources are shown in Figure 4(a). The photon densities of the sources are shown in Figure 5(a). The quantitative data on the reconstruction are listed in Table 2.

### 3.3. Multiband reconstruction

We performed the proposed multispectral BLT reconstruction using the same numerical model described in Section 3.2. According to the literature [19, 20], various optical parameters (absorption, scattering) were assigned to different regions of the numerical heterogeneous phantom, as listed in Table 3.

Three measurement data sets on the 1024 detector points on the phantom side surface were similarly generated for spectral ranges [400 nm, 530 nm], [530 nm, 630 nm], and [630 nm, 750 nm], respectively. The data sets were also corrupted with 20% Gaussian noise. With the same definition for the stabilizing function and the regularization parameter

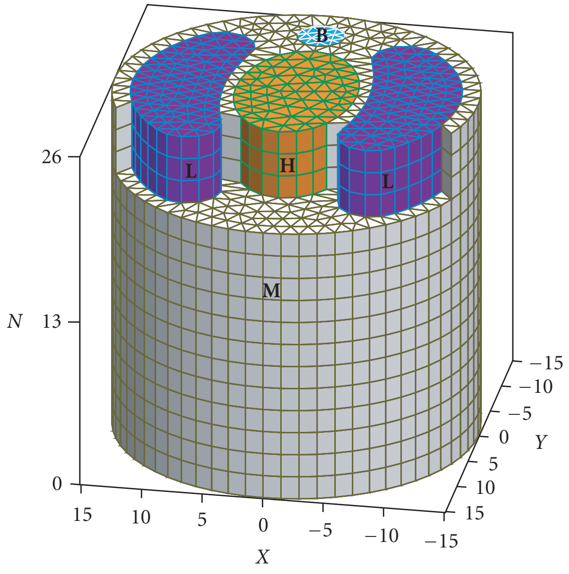

(a)

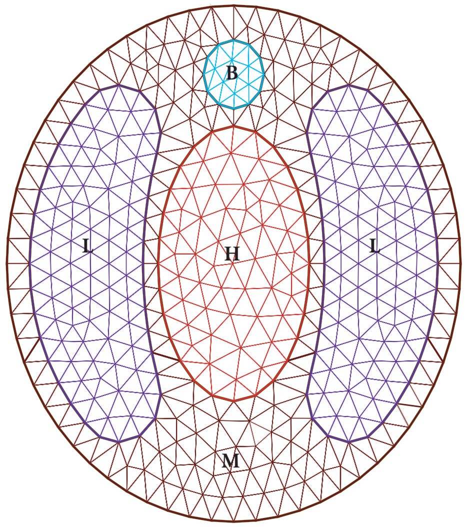

(b)

Figure 2: Heterogeneous mouse chest phantom with regions geometrically similar to lungs (L), heart (H), bone (B), and muscle (M), respectively. (a) The geometric phantom, and (b) the middle cross-section of the phantom.

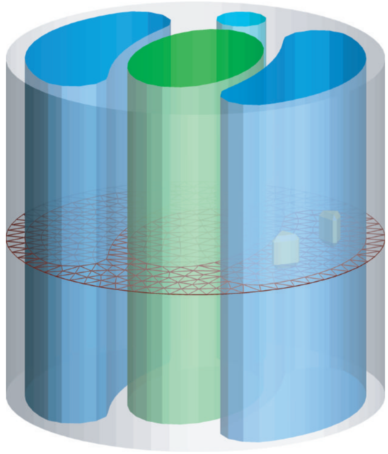

(a)

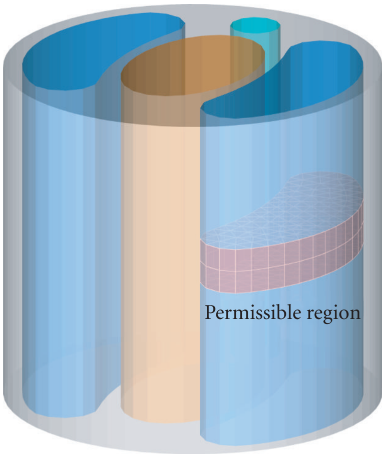

(b)

Figure 3: Representative source distribution. (a) Actual source positions, and (b) the selected permissible region.

Table 1: Optical parameters of each component in the heterogeneous phantom.

| Region | $\mu_a(\mathrm{mm}^{-1})$ | $\mu_s'(\mathrm{mm}^{-1})$ |
|---|---|---|
| Muscle | 0.0068 | 1.081 |
| Lung | 0.0233 | 1.974 |
| Heart | 0.0104 | 1.008 |
| Bone | 0.0001 | 0.060 |

in Section 3.2, the three simulated data sets were taken into the multiband/multispectral reconstruction of the single-source distribution using our method described in Section 2. The reconstructed locations of the sources are shown in Figure 4(b). The photon densities of the sources are shown in Figure 5(b). The multiband reconstruction is quantitatively compared to the single-band reconstruction in Table 2. The reconstructed results of multispectral BLT are clearly superior to those of single-spectral BLT.

### 4. DISCUSSIONS AND CONCLUSION

It seems that the multispectral approach is both feasible and beneficial. Target cells can now be tagged with reporters encoding several kinds of luciferase enzymes, resulting in a much broader emission spectrum. Dichroic filters can be used to separate the mixed signal into several wavelength

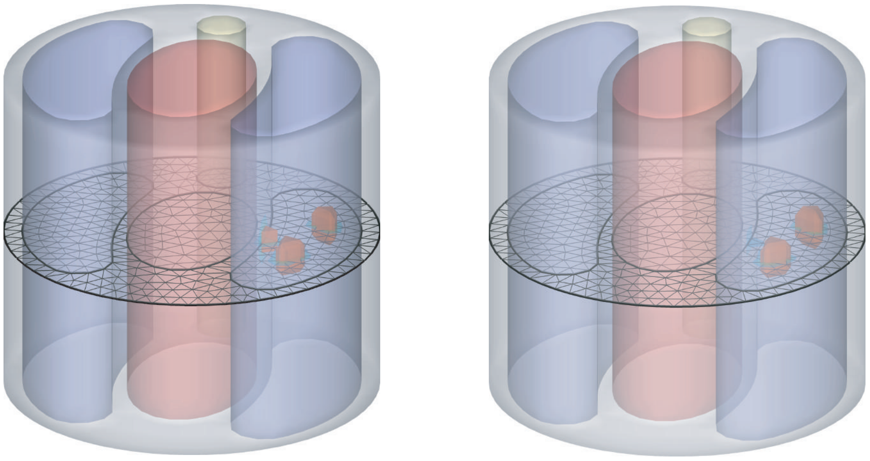

(a) (b)

Figure 4: Single-band and multispectral reconstructions. (a) The source distribution reconstructed using the single-band algorithm, and (b) the counterpart reconstructed using the multispectral algorithm.

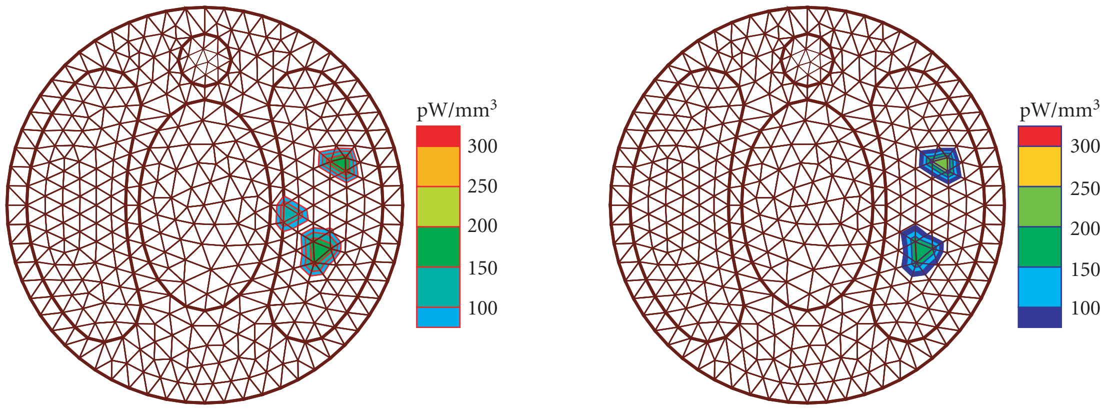

(a) (b)

Figure 5: Source densities in the single-band and multispectral reconstructions. (a) The source density reconstructed using the single-band algorithm, and (b) the counterpart reconstructed using the multispectral algorithm.

Table 2: Reconstruction results obtained using the single-band and multiband algorithms.

| Source | Single-band reconstruction | | Multiband reconstruction | |
|---|---|---|---|---|
| | Photon density (pW/mm$^3$) | Relative error | Photon density (pW/mm$^3$) | Relative error |
| Source | 183.06 | 39.0% | 195.69 | 34.8% |
| Source | 194.58 | 35.1% | 236.12 | 21.3% |

bands. As a result, a much more effective measurement can be made to improve the BLT reconstruction of such a source distribution. This multiband method significantly reduces the ill-posedness of the BLT problem. Our simulation results have showed that the multiband reconstruction has a better accuracy than the single-band reconstruction, especially in the case of high noise.

In conclusion, we have presented the multiband reconstruction method for BLT based on finite-element analysis. The multispectral approach greatly increases the measurement information, effectively reduces the ill-posedness of BLT, and significantly improves the accuracy and stability of the bioluminescent source reconstruction. Physical phantom and in vivo small animal experiments will be performed in the future. Also, we are investigating the multispectral BLT in the second sense discussed in the first section.

Table 3: Optical parameters for each component in the phantom in every band of interest.

| Region | Wavelength (630 nm–750 nm) $\mu_a(\text{mm}^{-1})$ | $\mu_s'(\text{mm}^{-1})$ |
|---|---|---|
| Muscle | 0.0052 | 1.081 |
| Lung | 0.0103 | 1.974 |
| Heart | 0.0078 | 1.008 |
| Bone | 0.0001 | 0.060 |
| Region | Wavelength (530 nm–630 nm) $\mu_a(\text{mm}^{-1})$ | $\mu_s'(\text{mm}^{-1})$ |
| Muscle | 0.0068 | 1.031 |
| Lung | 0.0233 | 1.880 |
| Heart | 0.0104 | 0.986 |
| Bone | 0.0001 | 0.060 |
| Region | Wavelength (400 nm–530 nm) $\mu_a(\text{mm}^{-1})$ | $\mu_s'(\text{mm}^{-1})$ |
| Muscle | 0.0088 | 1.001 |
| Lung | 0.0423 | 1.833 |
| Heart | 0.0300 | 0.954 |
| Bone | 0.0001 | 0.060 |

### ACKNOWLEDGMENTS

This work is supported by NIH/NIBIB Grant EB001685. The authors are grateful to Drs. Durairaj Kumar, Wenxiang Cong, Ming Jiang, Eric Hoffman, and Yue (Joseph) Wang for discussions.

### REFERENCES

[1] S. Bhaumik and S. S. Gambhir, "Optical imaging of *Renilla* luciferase reporter gene expression in living mice," *Proceedings of the National Academy of Sciences of the United States of America*, vol. 99, no. 1, pp. 377–382, 2002.

[2] C. H. Contag and M. H. Bachmann, "Advances in in vivo bioluminescence imaging of gene expression," *Annual Review of Biomedical Engineering*, vol. 4, pp. 235–260, 2002.

[3] P. Ray, A. M. Wu, and S. S. Gambhir, "Optical bioluminescence and positron emission tomography imaging of a novel fusion reporter gene in tumor xenografts of living mice," *Cancer Research*, vol. 63, no. 6, pp. 1160–1165, 2003.

[4] H. Zhao, T. C. Doyle, O. Coquoz, F. Kalish, B. W. Rice, and C. H. Contag, "Emission spectra of bioluminescent reporters and interaction with mammalian tissue determine the sensitivity of detection in vivo," *Journal of Biomedical Optics*, vol. 10, no. 4, pp. 041210/1–041210/9, 2005.

[5] Y. Nakajima, M. Ikeda, T. Kimura, S. Honma, Y. Ohmiya, and K.-I. Honma, "Bidirectional role of orphan nuclear receptor ROR$\alpha$ in clock gene transcriptions demonstrated by a novel reporter assay system," *FEBS Letters*, vol. 565, no. 1–3, pp. 122–126, 2004.

[6] B. W. Rice, M. D. Cable, and M. B. Nelson, "In vivo imaging of light-emitting probes," *Journal of Biomedical Optics*, vol. 6, no. 4, pp. 432–440, 2001.

[7] A. D. Klose, V. Ntziachristos, and A. H. Hielscher, "The inverse source problem based on the radiative transfer equation in optical molecular imaging," *Journal of Computational Physics*, vol. 202, no. 1, pp. 323–345, 2005.

[8] S. R. Arridge, M. Schweiger, M. Hiraoka, and D. T. Delpy, "A finite element approach for modeling photon transport in tissue," *Medical Physics*, vol. 20, no. 2, pt 1, pp. 299–309, 1993.

[9] T. J. Farrell, M. S. Patterson, and B. Wilson, "A diffusion theory model of spatially resolved, steady-state diffuse reflectance for the noninvasive determination of tissue optical properties in vivo," *Medical Physics*, vol. 19, no. 4, pp. 879–888, 1992.

[10] M. Gurfinkel, T. Pan, and E. M. Sevick-Muraca, "Determination of optical properties in semi-infinite turbid media using imaging measurements of frequency-domain photon migration obtained with an intensified charge-coupled device," *Journal of Biomedical Optics*, vol. 9, no. 6, pp. 1336–1346, 2004.

[11] M. Guven, B. Yazici, X. Intes, and B. Chance, "Diffuse optical tomography with *a priori* anatomical information," *Physics in Medicine and Biology*, vol. 50, no. 12, pp. 2837–2858, 2005.

[12] G. Wang, E. A. Hoffman, G. McLennan, L. V. Wang, M. Suter, and J. Meinel, "Development of the first bioluminescent CT scanner," *Radiology*, vol. 229(P), p. 566, 2003.

[13] G. Wang, E. A. Hoffman, and G. McLennan, Systems and methods for bioluminescent computed tomographic reconstruction. Patent disclosure filled in July 2002; US provisional patent application filled in March 2003; US patent application filed in March 2004.

[14] G. Wang, Y. Li, and M. Jiang, "Uniqueness theorems in bioluminescence tomography," *Medical Physics*, vol. 31, no. 8, pp. 2289–2299, 2004.

[15] W. Cong, D. Kumar, Y. Liu, A. Cong, and G. Wang, "A practical method to determine the light source distribution in bioluminescent imaging," in *Developments in X-Ray Tomography IV*, vol. 5535 of *Proceedings of SPIE*, pp. 679–686, Denver, Colo, USA, August 2004.

[16] X. Gu, Q. Zhang, L. Larcom, and H. Jiang, "Three-dimensional bioluminescence tomography with model-based reconstruction," *Optics Express*, vol. 12, no. 17, pp. 3996–4000, 2004.

[17] M. Jiang, G. Wang, and Y. Li, "Inverse problems in bioluminescence tomography," in *Frontier and Prospect of Contemporary Applied Mathematics*, P. G. Ciarlet and T. Li, Eds., Series in Contemporary Applied Mathematics, Higher Education Press; World Scientific, Beijing, China, 2005.

[18] W. Cong, G. Wang, D. Kumar, et al., "Practical reconstruction method for bioluminescence tomography," *Optics Express*, vol. 13, no. 18, pp. 6756–6771, 2005.

[19] C. Kuo, O. Coquoz, D. G. Stearns, and B. W. Rice, "Diffuse luminescence tomography of in vivo bioluminescent markers using multi-spectral data," in *Proceedings of the 3rd Annual Meeting of the Society for Molecular Imaging*, vol. 3, p. 227, St. Louis, Mo, USA, September 2004, abstract no. 180.

[20] G. Alexandrakis, F. R. Rannou, and A. F. Chatziioannou, "Tomographic bioluminescence imaging by use of a combined optical-PET (OPET) system: a computer simulation feasibility study," *Physics in Medicine and Biology*, vol. 50, no. 17, pp. 4225–4241, 2005.

[21] J. J. Duderstadt and L. J. Hamilton, *Nuclear Reactor Analysis*, John Wiley & Sons, New York, NY, USA, 1976.

[22] M. Schweiger, S. R. Arridge, M. Hiraoka, and D. T. Delpy, "The finite element method for the propagation of light in scattering media: boundary and source conditions," *Medical Physics*, vol. 22, no. 11, pt 1, pp. 1779–1792, 1995.

[23] S. S. Rao, *The Finite Element Method in Engineering*, Butterworth-Heinemann, Boston, Mass, USA, 1999.

[24] P. P. B. Eggermont, "Maximum entropy regularization for Fredholm integral equations of the first kind," *SIAM Journal on Mathematical Analysis*, vol. 24, no. 6, pp. 1557–1576, 1993.

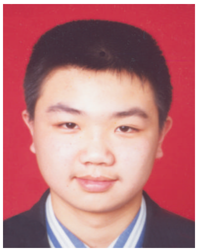

**Alexander X. Cong** is studying electrical and computer engineering in the University of Toronto and currently working as a Research Assistant at the Center for X-Ray and Optical Tomography, University of Iowa. His scientific interests are in the fields of bioluminescence tomography, fluorescence tomography, and medical imaging.

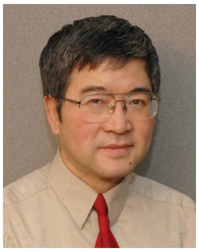

**Ge Wang** received the B.E. degree in electrical engineering from Xidian University, Xian, China, in 1982, the M.S. degree in remote sensing from the Graduate School of Academia Sinica, Beijing, China, in 1985, and the M.S. and Ph.D. degrees in electrical and computer engineering from the State University of New York, Buffalo, in 1991 and 1992. He was an Instructor and Assistant Professor with the Department of Electrical Engineering, Graduate School of Academia Sinica, in 1984–1988, and Instructor and Assistant Professor with Mallinckrodt Institute of Radiology, Washington University, St. Louis, Mo, in 1992–1996. He was an Associate Professor with the University of Iowa in 1997–2002. Currently, he is a Professor with the Departments of Radiology, Biomedical Engineering, Mathematics, Civil Engineering, and Electrical and Computer Engineering, and Director of the Center for X-Ray and Optical Tomography, University of Iowa. His interests include computed tomography, bioluminescence tomography, and systems biomedicine. He has published over 300 journal articles and conference papers, including the first paper on spiral/helical cone-beam CT and the first paper on bioluminescence tomography. He is the Editor-in-Chief for the International Journal of Biomedical Imaging, and an Associate Editor for the IEEE Transactions on Medical Imaging and the Journal of Medical Physics. He is an IEEE Fellow and an AIMBE Fellow. He is also recognized by a number of awards for academic achievements.

